# Post-Traumatic Stress Disorder and participation in daily life: The pilot study of participation patterns and affecting factors

**DOI:** 10.1192/j.eurpsy.2023.1016

**Published:** 2023-07-19

**Authors:** L. Lipskaya-Velikovsky, R. Shapira, Y. J. Baris Ginat

**Affiliations:** 1 The School of Occupational Therapy, Faculty of Medicine, The Hebrew University; 2 Intensive Day Care, The Jerusalem Mental Health Center, Jerusalem, Israel

## Abstract

**Introduction:**

Post-Traumatic Stress Disorder (PTSD), with prevalence of 14%, causes disability and burden to the person, his/her close environment and whole society due to, among other factors, interruption in a range of daily life activities. To date little research was done to delineate comprehensive patterns of daily life participation among people with PTSD. Despite extensive research, our understanding of factors affecting the participation in PTSD is limited, given that relief in the PTSD symptoms does not guarantee returning to satisfying daily life activities.

**Objectives:**

Investigate objective and subjective participation dimensions among individuals with PTSD in comparison to healthy controls; and explore the impact of personal and illness-related factors, body functions and environment on the participation in PTSD.

**Methods:**

Sixty two individuals with PTSD (age: M=34.3, SD =9.2; women: 24, 77.4%) and matching by age and gender healthy controls participated in this cross-sectional study. They completed standard assessments for PTSD symptoms severity, general cognitive profile, executive functions (EF) based on self-report and performance, sensory processing, self-efficacy, capacity to perform everyday activities, environmental properties, and actual participation in daily life in objective (number of activities, frequency, location and with whom) and subjective (enjoyment, satisfaction, meaningfulness) dimensions.

**Results:**

The participation was found to be inferior in PTSD in the following dimensions: number of activities, participation frequency, and enjoyment (2.72<3.9, p<.01), and experienced low meaning within the participation. Number of participated activities was correlated with self-reported EF (r=0.465, p<.05), and environment properties (r=0.5, p<.01). Frequency of participation was associated with self-reported EF (r=0.45, p<.05). In addition, number of activities, frequency and experience of meaning were inferior in those who reported on avoidance from sensory stimuli in daily life (71%; 2.5<t<2.9, p<.05). PTSD symptoms severity was not correlated with the participation (-0.35<r<-0.01, p<.05).

**Conclusions:**

The restriction in both objective and subjective dimensions of participation in PTSD raises major concern given the profound effect of participation on well-being, and individual and community burden. The study reveals unique patterns of association between the participation indices and personal and illness related factors in PTSD, suggesting that objective factors are of less impact in comparison to subjective ones; and aspects of cognitive and sensory regulation as well as environment are of particular importance for participation. This pilot study demonstrates a need for further research to expand our knowledge in the field with the ultimate goal of contributing to well-being and health of individuals with PTSD.

**Disclosure of Interest:**

None Declared

